# Exploring the binding of BACE-1 inhibitors using comparative binding energy analysis (COMBINE)

**DOI:** 10.1186/1472-6807-12-21

**Published:** 2012-08-27

**Authors:** Shu Liu, Rao Fu, Xiao Cheng, Sheng-Ping Chen, Li-Hua Zhou

**Affiliations:** 1 Guangdong Province Key Laboratory of Functional Molecules in Oceanic Microorganism, Zhong Shan School of Medicine, Sun Yat-Sen University, Guangzhou, 510080, People’s Republic of China; 2 Department of Anatomy, Zhong Shan School of Medicine, Sun Yat-Sen University, Guangzhou, 510080, People’s Republic of China

**Keywords:** BACE-1 Inhibitors, Superimposition, 3D-QSAR, COMBINE

## Abstract

**Background:**

The inhibition of the activity of β-secretase (BACE-1) is a potentially important approach for the treatment of Alzheimer disease. To explore the mechanism of inhibition, we describe the use of 46 X-ray crystallographic BACE-1/inhibitor complexes to derive quantitative structure-activity relationship (QSAR) models. The inhibitors were aligned by superimposing 46 X-ray crystallographic BACE-1/inhibitor complexes, and gCOMBINE software was used to perform COMparative BINding Energy (COMBINE) analysis on these 46 minimized BACE-1/inhibitor complexes**.** The major advantage of the COMBINE analysis is that it can quantitatively extract key residues involved in binding the ligand and identify the nature of the interactions between the ligand and receptor.

**Results:**

By considering the contributions of the protein residues to the electrostatic and van der Waals intermolecular interaction energies, two predictive and robust COMBINE models were developed: (i) the 3-PC distance-dependent dielectric constant model (built from a single X-ray crystal structure) with a q^2^ value of 0.74 and an SDEC value of 0.521; and (ii) the 5-PC sigmoidal electrostatic model (built from the actual complexes present in the Brookhaven Protein Data Bank) with a q^2^ value of 0.79 and an SDEC value of 0.41.

**Conclusions:**

These QSAR models and the information describing the inhibition provide useful insights into the design of novel inhibitors via the optimization of the interactions between ligands and those key residues of BACE-1.

## Background

It is generally accepted that Alzheimer’s disease (AD) is caused by extracellular amyloid plaque deposition and the intracellular formation of neurofibrillary tangles in the brain [1-4]. β-amyloid peptides (Aβ, forming the amyloid plaques) are formed by the action of the β-secretase (BACE-1) and γ-secretase enzymes on the amyloid precursor protein (APP) [5-8]. BACE-1 is currently widely accepted as a leading target for the therapeutic treatment of AD [9-12]. The inhibition of BACE-1 can prevent the cleavage of APP to Aβ and the formation of amyloid plaques [13].

The search for potent BACE-1 inhibitors is being pursued actively in many academic institutes and pharmaceutical companies. Most of these endeavors include computational studies such as pharmacophore modeling [14,15], classical quantitative structure-activity relationships (QSARs) [14-17], docking and virtual screening [18-22] and molecular dynamics (MD) simulations [23-26]. Currently, several hundred BACE-1 inhibitors have been reported, but most of these inhibitors are peptidomimetics [16]. To find novel BACE-1 inhibitors, a few companies are actively screening against BACE-1. A research group from Merck has performed in vitro high-throughput screening (HTS) and found a single molecule (a 1,3,5-trisubstituted benzene) as a hit from a multi-million compound library [27], whereas Astex Therapeutics has taken a fragment-based lead generation approach [28]. After the virtual screening of a fragment library, a small number of potential structures were soaked with BACE-1 crystals in anticipation of obtaining a co-crystal with the enzyme. Johnson & Johnson Pharmaceutical R&D also reported a novel cyclic guanidine screening lead; the initial screening lead had an IC_50_ value of 900 nM [29]. Huang et al. performed in silico screening of 180,000 small chemicals and found 10 diacylurea inhibitors that exhibited an IC_50_ value lower than 100 μM in an enzymatic assay. Four of these inhibitors were cell penetrant (EC_50_ < 20 μM) [21].

3D-QSAR studies are very helpful in the design of novel lead compounds. Zuo et al. explored the binding mechanism of 32 statine-based peptidomimetic inhibitors of BACE-1 using CoMFA (comparative molecular field analysis) and CoMSIA (comparative molecular similarity indices analysis) methods. Based on molecular docking results, 3D-QSAR models were developed with q^2^ values of 0.582 and 0.622 using CoMFA and CoMSIA, respectively [17]. A study of the mechanism of the interaction between BACE-1 and its inhibitors would be valuable in discovering more active drug-like inhibitors that block the function of BACE-1. To glean critical information regarding the interactions of the inhibitors with the residues in the active site of BACE-1, we conducted a 3D-QSAR study of 46 BACE-1/inhibitor complexes using the COMparative BINding Energy (COMBINE) method. The COMBINE method, first developed by A. R. Ortiz in 1995 [30], has been widely applied in the field of drug design [31-37]. In 2010, Gil-Redondo et al. developed gCOMBINE [38], a Java graphical user interface (GUI), to perform COMBINE analyses, providing a convenient tool for academic researchers. The key idea of COMBINE analysis is that a simple expression describing the differences in binding affinity of a series of related ligand-receptor complexes can be derived by using multivariate statistics to correlate experimental data on binding affinities with components of the ligand-receptor interaction energy computed from energy-minimized 3D structures. Some other forms of free energy calculations, such as MM-PBSA, MM-GBSA [39], or linear interaction energy (LIE) simulation [40], use Monte Carlo, or molecular dynamics simulations to calculate the protein-ligand interaction energies. However, COMBINE analysis only needs static structures and this approach can reduce the computational burden. Compared with the classical 3D-QSAR methods (CoMFA and CoMSIA) [41], COMBINE analysis can aid researchers in acquiring quantitative or semiquantitative insight into the key role played by specific protein-ligand interactions and/or desolvation components. As a result, residue-based van der Waals and electrostatic contributions that are endowed with a higher discriminatory ability can be identified, which provides clues for further chemical modification throughout the series. It has also been demonstrated that regression models derived with COMBINE analysis are suitable for fast virtual screening of compound databases [37].

Alignment is a crucial component in 3D-QSAR studies, and many researchers have used docking methods to align ligands when 3D protein structures were available [17]. However, due to various approximations and trade-offs involved in the formulations because of the computational cost, the current scoring functions are unable to accurately assess the ligand binding poses. To overcome this disadvantage of the dock methods, in the present study, we replaced the docking method with a superimposing X-ray protein/inhibitor complex method to align the ligands. It has been eleven years since the first BACE-1 crystal structure was reported. Currently, there are more than 150 X-ray crystal structures of BACE-1/inhibitor complexes in the Brookhaven Protein Data Bank (PDB) [42]. Taking into consideration the diversity of the inhibitors, we chose 46 crystal structures of BACE-1/inhibitor complexes from the Brookhaven PDB. Using a COMBINE analysis, we obtained a robust COMBINE model, which should be useful for understanding the inhibitory mode of BACE-1 and in designing novel lead compounds against Alzheimer’s disease.

## Results and discussion

In this study, choosing the 1 W51 structure as the reference for all the alignments was based on previous research [43]. Polgar and Keseru have performed a comparative virtual screen for BACE-1 inhibitors using different protein conformations (1SGZ, 1FKN, 1 W51, 1XS7 and 1M4H). In that study, the use of 1 W51 as a target gave the best enrichment factors and the ligands found proper poses more easily [44]. Furthermore, in our previous studies, by comparing different reference structures, we found that the use of the 1 W51 structure gave better binding affinity models [43]. Therefore, despite the availability of other crystal structures of BACE-1 serving as the reference structure, we concluded that the 1 W51 structure represented the most suitable reference structure.

**Figure 1:**
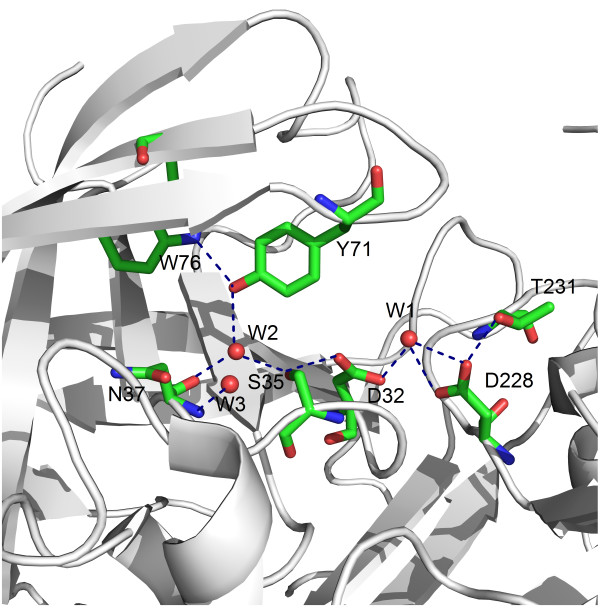
**The catalytic site of BACE-1 (1TQF structure).** The Thr231 hydroxyl group located at a hydrogen bond distance from the Asp228 carboxyl moiety protects this group from protonation. A conserved water molecule (W2), forms three hydrogen bonds with residues Tyr71, Asn37 and Ser35, resulting in the formation of a continuous chain of hydrogen-bonded residues Trp76-Tyr71-W2-Ser35-Asp32 that connect the flap with the catalytic site. Hydrogen bonds are shown by dashed lines, amino acids are shown by sticks and water molecules are shown by red balls.

By a standard superposition technique [45], we analyzed and compared 46 crystal structures to explore the protonation state of the catalytic Asp residues, and to clarify the functional role and stability of the two conserved water molecules (W1 and W2) in the BACE-1 active site (Figure 1). The catalytic water (W1) bridges the two catalytic aspartate residues and is involved in nucleophilic attack. Structural data suggest the average distance between the carboxyl oxygens of the catalytic Asp dyad is 2.5–3.5 Å for all 46 complexes (Figure 1), it indicates that hydrogen bonding between Asp32 and Asp228 is possible in the presence of a substrate. The location of the Thr231 hydroxyl group at a hydrogen bond distance from the Asp 228 carboxyl is a common feature of the BACE-1 complexes with inhibitors. This phenomenon was supported by the reaction mechanism proposed by Andreeva and Rumsh, i.e., Thr231 protects the Asp228 carboxyl from protonation [46].

At the same time, the proximity of the Ser35 hydroxyl group to the Asp32 carboxyl group found in BACE-1 was observed in all complexes with inhibitors. It is important to note that another water molecule (W2), in the vicinity of the active groups, is completely conserved [46]. This water molecule forms a hydrogen bond with side-chain hydroxyl of Ser35 and this kind of interaction was observed in all analyzed structures. Besides Ser35, W2 is oriented such that it acts as a donor to the Asn37 backbone carbonyl. This bond is also conserved. W2 also forms a third hydrogen bond with the hydroxyl of the conserved residue Tyr71; the Tyr71 hydroxyl acts as an acceptor for the NH of Trp76. These interactions form a continuous chain of hydrogen-bonded residues, Trp76-Tyr71-W2-Ser35-Asp32, thereby connecting the flap with the catalytic site. Structural data suggest the existence of a mechanism that assists releasing a proton from the Asp32 carboxyl during the initial stages of catalysis, and acceptance of a proton after substrate cleavage. This mechanism arises from the ability of the Ser35 hydroxyl and the water molecule W2 to exchange their donor and acceptor roles while being hydrogen-bonded. In other word, Ser35 assists in proton acceptance and release of Asp32 during the catalytic cycle.

The identification of the protonation states of the key aspartate residues in BACE-1 is of significant interest both in understanding the reaction mechanism and in guiding the design of drugs against Alzheimer’s disease. However, researchers have not reached consensus on the experimental and theoretical studies of the aspartate protonation states [20,26,47,48]. By structural data analysis, our results are consistent with the results of Polgar and Keseru [20]. Polgar and Keseru performed pKa calculations to study the protonation state of catalytic Asp residues (Asp32, Asp228) of BACE-1 based on the finite-difference solution of the Poisson-Boltzmann equation. Their research concluded that crystals of BACE-1 (1SGZ, 1FKN) were grown at pH 6.5 and 7.4 and under this condition only the Asp228 residue is ionized (Asp32, Asp228^−^).

In addition, tautomerism could influence the results of the COMBINE analysis. Tautomerism, which is a phenomenon whereby a compound interconverts to other isomers that differ in the position of a double bond and one atom (typically a hydrogen atom), is of special interest in studies of protein-ligand interactions [49]. Since the displacement of a hydrogen atom may convert an acceptor into a donor, a tautomeric rearrangement changes the interaction landscape of a protein-ligand complex. In this study, we should initially examine whether there are tautomers among these 46 co-crystallized ligands. Next, we had to estimate the preferred tautomer in the binding site for each tautomer by analyzing the hydrogen bond interactions. This is because the positions of the hydrogen atoms in the PDB structures were not determined due to the resolution limits of the structures. By visual inspection, some tautomeric structures among these 46 co-crystallized ligands were found and the main tautomeric forms can be represented as amide-imidic acid and allyl amine-imine. By analyzing the structural data, the most favorable hydrogen bond interactions were identified. Table 1 summarizes the most preferred tautomer in the binding site for each compound. Moreover, according to the above analysis the protonation state of BACE-1 was assessed as Asp32^+^ and Asp228^−^. Therefore, this protonation state (Asp32^+^ and Asp228^−^) and the most preferred tautomer of each co-crystallized inhibitor were applied in the following COMBINE analysis.

**Table 1:** Data set of the 46 co-crystallized ligands of BACE-1

		
^a^1. 1W51, L01	2. 1TQF, 32P	3. 1YM2, AUA
		
4. 1YM4, AMK	5. 2B8V, 3BN	6. 2F3E, AXQ
		
7. 2F3F, AXF	8. 2IQG, F2I	9. 2IRZ, I02
		
10. 2IS0, I03	11. 2OAH, QIN	12. 2OHL, 2AQ
		
13. 2OHM, 8AP	14. 2OHP, 6IP	15. 2OHQ, 7IP
		
16. 2OHR, 8IP	17. 2OHS, 9IP	18. 2OHT, IP6
		
19. 2OHU, IP7	20. 2P83,MR0	21. 2PH6, 712
		
22. 2B8L, 5HA	23. 2QZL, IXS	24. 2ZE1, 411
		
25. 2QP8, SC7	26. 2VIE, VG0	27. 2VJ7, VG6
		
28. 2VJ9, VG7	29. 2VNM, CM8	30. 2VNN, CM7
		
31. 2WF0, ZY0	32. 2WF1, ZY1	33. 2ZDZ, 310
		
34. 2FDP, FRP	35. 3CIB, 314	36. 3CIC, 316
		
37. 3CID, 318	38. 3DM6, 757	39. 3DUY, AFJ
		
40. 3DV1, AR9	41. 2P4J, 23I	42. 3FKT, SII
		
43. 2QK5, CS5`	44. 1XS7, MMI	
		
45. 1FKN, OM99-2	46. 1M4H, OM00-3	

**Figure 2:**
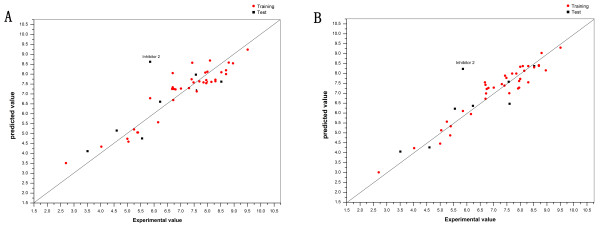
**Scatter plot comparing experimental vs. predicted activities in COMBINE models for the 46 compounds of the training series and the test series.A.** The 3-PC distance-dependent dielectric constant model (built from a single X-ray crystal structure). **B.** The 5-PC sigmoidal electrostatic model (built from the actual complexes present in the PDB).

In present study, three types of electrostatic models (Model **1:** a distance-dependent dielectric constant model [50]; Model **2:** a uniform dielectric constant model [51]; and Model **3:** a sigmoidal model [50]) were used. The q^2^ value served as the criterion to determine the optimal dimensionality of the PLS models. The standard deviation of errors of correlation (SDEC) value for the 38 internal training sets and the average standard deviation of errors of prediction (SDEP) value for the eight external test sets are listed in Table 2. To justify the docked conformation of the inhibitors from their respective complexes, root-mean-square deviation (RMSD) was used as a good measure to evaluate the predicted power of a docking result It is generally accepted that a successful docking result reproduces the crystallographic conformation of a ligand in the complex structure within a 2 Å RMSD on all ligand atoms. Three protocols were performed to translocate the other 45 co-crystallized inhibitors to a single active pocket of BACE-1 (PDB entry 1 W51). Protocol 1, energy minimization after protein superposition; Protocol 2: energy minimization before protein superposition; Protocol 3: docking by Surflex. Subsequently, we performed a COMBINE analysis of the 46 BACE-1/inhibitor complexes (each inhibitor and the A chain of 1 W51). As indicated in Table 3 and judging from the RMSD value, protocol 1 reproduced the native crystallographic conformation to its fullest extent. As indicated in Table 2, among the three types of electrostatic models, we found that Model 1 outperformed Models 2 and 3, in which three latent variables (PCs) yielded an r^2^ of 0.87, a q^2^ of 0.74, and an SDEC value of 0.53. The SDEP value for the external validation was 1.13, as expected, which is larger than that for the internal validation but sufficient to demonstrate the robustness of the model. The predicted pIC_50_ values are listed in Table 3, and plotted against the experimental pIC_50_ values for the model with three latent variables in Figure 2A. We can see that this 3-PC COMBINE model produces more accurate predictions than those obtained in previous CoMFA and CoMSIA studies of BACE-1 inhibitors [17].

**Table 2:** **Performance of different COMBINE models**^**a**^**for the whole set of inhibitors in fitting and prediction**

		**Data set 1**^**b**^				**Data set 2**^**b**^			
**Electrostatic Models**^**c**^	**No. of PCs**	**r**^**2**^	**SDEC**	**q**^**2**^	**SDEP**	**r**^**2**^	**SDEC**	**q**^**2**^	**SDEP**
**1**	1	0.709	0.78	0.653	0.948	0.588	0.929	0.51	1.153
	2	0.82	0.614	0.713	0.914	0.793	0.659	0.701	0.816
	3	**0.871**	**0.521**	**0.74**	**1.131**	0.891	0.477	0.703	1.074
	4	0.917	0.417	0.713	0.989	0.91	0.435	0.761	1.096
	5	0.944	0.344	0.691	1.00	0.923	0.402	0.775	0.98
**2**	1	0.252	1.336	0.149	1.609	0.295	1.215	0.106	1.571
	2	0.718	0.994	0.528	1.116	0.78	0.679	0.704	0.913
	3	0.856	0.848	0.657	0.931	0.824	0.607	0.719	0.785
	4	0.898	0.805	0.691	1.097	0.896	0.466	0.747	1.06
	5	0.921	0.824	0.677	1.131	**0.92**	**0.41**	**0.786**	**0.988**
**3**	1	0.212	1.37	0.105	1.623	0.26	1.245	0.045	1.621
	2	0.773	0.845	0.66	0.85	0.79	0.663	0.725	0.949
	3	0.864	0.819	0.68	1.088	0.819	0.617	0.731	0.796
	4	0.922	0.889	0.623	1.007	0.897	0.465	0.728	0.728
	5	0.956	1.002	0.521	0.87	0.916	0.419	0.772	0.816

^a^Abbreviations: r^2^, correlation coefficient; SDEC, standard deviation of errors of correlation; q^2^, predictive correlation coefficient; SDEP, standard deviation of errors of prediction.

^b^Data set 1, all the BACE-1/inhibitor complexes were built from the A chain of 1 W51 and each of the co-crystallized ligands. All the co-crystallized ligands were translocated directly to the binding pocket of 1 W51 by a superimposition method using the C_α_ atoms (1 W51 structure as the reference), after this process, each BACE-1/inhibitor complex was energy minimized using the AMBER 9.0 program.

Data set 2, all the BACE-1/inhibitor complexes were the actual complexes present in the PDB and energy minimized with the AMBER 9.0 program.

^c^The three types of electrostatic models. Model **1:** a distance-dependent dielectric constant model. Model **2:** a uniform dielectric constant model. Model **3:** a sigmoidal model. The values in bold highlight the best quality models.

**Table 3:** The RMSD values of three alignment protocols

**Compound Number**	**Pdb ID**	**Ligand**	**RMSD(Å)**^**a**^	**RMSD(Å)**^**b**^	**RMSD(Å)**^**c**^	**pIC**_**50**_^**d**^	**pIC**_**50**_^**e**^	**pIC**_**50**_^**f**^
1	1 W51	L01	0.36	0.36	0.645	6.7	7.337	6.72
2	1TQF	32P	0.328	0.409	3.608	5.857	8.627	8.23
3	1YM2	AUA	0.38	0.348	0.95	8.0	7.617	8.34
4	1YM4	AMK	0.305	0.485	2.24	7.41	7.74	7.38
5	2B8V	3BN	0.416	0.47	1.739	7.01	7.27	7.28
6	2F3E	AXQ	0.27	0.242	0.676	6.81	7.23	7.26
7	2F3F	AXF	0.27	0.344	1.445	6.72	6.69	6.98
8	2IQG	F2I	0.398	0.404	0.846	8.3	7.72	7.55
9	2IRZ	I02	0.401	0.354	2.219	7.92	8.08	7.24
10	2IS0	I03	0.483	0.556	2.184	6.7	8.05	7.42
11	2OAH	QIN	0.857	0.525	2.145	7.96	7.7	7.61
12	2OHL	2AQ	0.144	0.192	0.08	2.7	3.51	3.01
13	2OHM	8AP	0.36	0.468	1.524	3.51	4.12	4.06
14	2OHP	6IP	0.576	0.535	2.432	4.03	4.34	4.22
15	2OHQ	7IP	0.35	0.501	1.29	4.6	5.15	4.27
16	2OHR	8IP	0.297	0.359	2.428	5.0	4.74	4.45
17	2OHS	9IP	0.256	0.314	2.438	5.4	5.06	5.33
18	2OHT	IP6	0.455	0.464	1.743	5.04	4.6	5.13
19	2OHU	IP7	0.906	0.942	1.921	5.38	5.06	4.87
20	2P83	MR0	0.345	0.363	1.903	7.96	7.54	7.28
21	2PH6	712	0.371	0.563	3.184	7.57	7.97	7.58
22	2B8L	5HA	0.316	0.433	2.51	8.0	8.12	7.72
23	2QZL	IXS	0.41	0.4	2.122	8.1	8.68	8.37
24	2ZE1	411	0.825	0.851	0.946	5.25	5.21	5.56
25	2QP8	SC7	0.355	0.342	0.652	8.15	7.61	8.13
26	2VIE	VG0	0.396	0.52	1.705	7.48	7.58	7.77
27	2VJ7	VG6	0.765	0.692	2.0	7.31	7.3	7.46
28	2VJ9	VG7	0.413	0.475	1.094	6.74	7.25	7.21
29	2VNM	CM8	0.387	0.572	2.457	8.52	8.09	8.3
30	2VNN	CM7	0.366	0.333	1.58	8.7	8.0	8.38
31	2WF0	ZY0	0.51	0.527	1.133	6.68	7.25	7.54
32	2WF1	ZY1	0.377	0.361	2.342	8.7	8.19	8.41
33	2ZDZ	310	0.871	0.901	1.642	6.15	5.57	5.95
34	2FDP	FRP	0.415	0.428	2.18	7.59	7.13	7.0
35	3CIB	314	0.343	0.355	0.558	7.85	7.58	7.99
36	3CIC	316	0.308	0.291	2.565	8.52	7.62	8.37
37	3CID	318	0.336	0.356	0.816	8.3	7.64	8.37
38	3DM6	757	0.659	0.716	2.814	7.43	8.57	7.88
39	3DUY	AFJ	0.443	0.653	1.749	5.85	6.78	6.1
40	3DV1	AR9	0.267	0.251	0.608	6.23	6.61	6.37
41	2P4J	23I	0.614	0.645	4.611	8.96	8.55	8.15
42	3FKT	SII	0.596	0.723	1.749	5.55	4.76	6.22
43	2QK5	CS5	0.405	0.392	2.416	7.7	7.63	7.99
44	1XS7	MMI	0.628	0.604	1.37	7.59	7.2	6.47
45	1FKN	OM99-2	1.262	1.56	3.3	8.8	8.58	9.03
46	1M4H	OM00-3	1.137	1.68	3.9	9.51	9.24	9.3

^a^Protocol 1. The 46 co-crystallized ligands were aligned in the binding pocket of 1W51 by the superimposition method using the C_α_ atoms (1W51 structure as the reference). Subsequently, each BACE-1/inhibitor complex (each inhibitor and A chain of 1W51) was energy minimized using the AMBER 9.0 program.

^b^Protocol 2. The 46 co-crystallized ligands were refined before translocation and energy minimization that was adopted in protocol 1.

^c^Protocol 3. The 46 co-crystallized ligands were docked into 1W51 using the Surflex program (version 2.11), followed by the energy minimization routine mentioned above.

^d^experimental.

^e^predicted with the 3-PC distance-dependent dielectric constant model (built from a single X-ray crystal structure).

^f^predicted with the 5-PC sigmoidal electrostatic model (built from the actual complexes present in the PDB).

Table 3 shows that the RMSD values of some co-crystallized ligands by protocol 3 were greater than 2 Å compared with their native crystallographic conformation. Although protocol 2 (energy minimization before protein superposition) could reproduce the native crystallographic conformation as well as protocol 1, in general the results using protocol 1 were superior to the results obtained using protocol 2. In addition, by using protocol 2, the COMBINE model was developed with q^2^ values of 0.69 and SDEC values of 0.719 (a distance-dependent dielectric constant 3-PC model), which was not so good as that in the 3-PC model obtained from protocol 1.

It is worthwhile to note that a ligand docked within 2 Å of the crystallographic pose can give rise to interactions with active site residues that are different from those found in the original X-ray crystal structures. Therefore, we performed a similar COMBINE analysis using the complexes present in the PDB and subjecting these complexes to a similar energy refinement mentioned above. In order to ensure the feasibility of performing the COMBINE analysis, the 1 W51 structure was set as the reference structure and all crystal structures were superimposed using the C_α_ atoms. In cases where the number of amino acids differed between complexes, we normalized all the crystal structures using a common number of residues. As indicated in Tables 2 and 3, the 4-PC or 5-PC COMBINE models generated from the complexes present in the PDB, was superior to the models built from the three protocols we used, regardless of which type of electrostatic model was applied. Table 2 shows that the 5-PC sigmoidal electrostatic model was the best, which yielded an r^2^ of 0.92, a q^2^ of 0.79 and an SDEC value of 0.41. The SDEP value for the external validation was 0.99. The predicted pIC_50_ values are presented in Table 3 and plotted against the experimental pIC_50_ values for the model with five latent variables in Figure 2B.

We are not surprised by the above results. A possible reason for these observations is that the application of the actual protein crystals was always considered to be more reliable than the artificial docking method. In addition, when building the COMBINE models with actual protein crystals, consideration for the effects of two or three water molecules in the catalytic site did improve the accuracy of the predictions. In comparison to the approach used, e.g., the COMBINE model built from a single X-ray crystal structure (1W51), the method using the actual complexes in the PDB was similar to the flexible docking approach, in which the effects of side-chains of several residues were considered.

As depicted in Figure 2A and B, both the 3-PC model (built from a single X-ray crystal structure) and 5-PC model (built from the actual complexes present in the PDB) behaved well for most of the compounds, However, the 1TQF ligand (inhibitor 2, co-crystallized ligand 32P [27]) was always determined to be an outlier in the COMBINE analysis, whether or not it was refined before being placed into the binding pocket of 1W51, or built from the actual complexes present in the PDB. Even when the water molecules in the catalytic site were taken into consideration, the 1TQF ligand was always an outlier, which differed by more than one order of magnitude between the predicted and experimental results. The primary reason was the different binding mode of the 1TQF ligand when compared with the binding modes of the other 45 ligands. In examining the enzyme-inhibitor complex (1TQF), the S4 to S1 subsites were found to be occupied by the inhibitor, and we did not observe any direct contact between the inhibitor and the catalytic aspartic dyad, Asp32 and Asp228. Instead, the inhibitor’s oxyacetamide NH moiety forms a hydrogen bond with a water molecule situated between the catalytic aspartic acids. Additionally, regarding the stereochemistry of the R-methyl-benzamide ligand, the structure reveals the presence of a novel S3 sub-pocket that binds the p-fluorophenyl ring.

To investigate the distribution of the 38 complexes (training set) in the space defined by their ligand-receptor interaction energies, a principal component analysis (PCA) was performed on the pretreated variables. As mentioned above, because there are 375 amino acids in the protein, and two energy contributions (van der Waals and electrostatic) were considered for each residue, an **X** matrix was built with 750 columns, representing each of these energy terms, and 38 rows representing each inhibitor in the series. A final column containing inhibitory activities is then added to the matrix. The X matrix was then transformed so that each column of data had an average of zero and a standard deviation of one. After removing those variables with a standard deviation below 0.01 kcal/mol, 49 residues were retained for the COMBINE interaction energy calculation, and 98 variables were selected to build the final PLS model. The dimensionality of the data matrix was reduced using a PCA method, while keeping the amount of information loss to a minimum. The number of latent variables (PCs) chosen for the model was that yielding the best cross-validated performance. The coefficients in a given PC provide information on the relative weight of the different terms and can be used to deduce the relevance of each individual ligand-residue interaction to explain the variance in activity.

From the point of view of statistical research, the first principal component (PC1) accounts for the maximum variance (eigenvalue) in the original dataset. The second principal component (PC2) is orthogonal (uncorrelated) to the first one, and it accounts for most of the remaining variance. This procedure is continued until the total variance is accounted for. The COMBINE analysis aims to tackle the X matrix with PCA analysis, and then use a multiple linear regression method to build a PLS model [31-37]. The q^2^ is a well-known indicator for evaluating the function of the number of principal components (PCs) extracted. Although using the 5-PC model (shown the on right side of Table 2 with bold type) for predicating the activity of the inhibitors gave a higher accuracy (a q^2^ value of 0.786), among all the 3-PC models, the distance-dependent dielectric constant 3-PC model (shown on the left side of Table 2 with bold type) was the top one, with a q^2^ value of 0.74. Selection of a smaller number of latent variables (PCs) chosen for the COMBINE model was more beneficial and simpler for the following research on identifying the nature of the interactions between the ligand and receptor. Such information is considered to be the most important factor that guides drug design. Therefore, in the next part of the discussion, we will focus on results calculated from the 3-PC model (shown on the left side of Table 2 with bold type).

**Figure 3:**
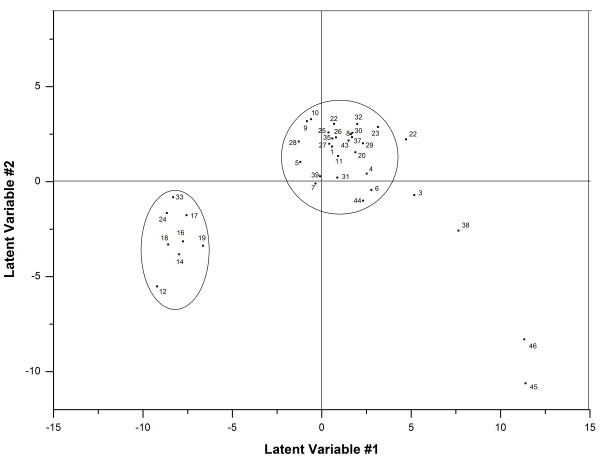
**Score plot of the first (PC1) and the second (PC2) principal components for COMBINE.** The relevant energy descriptors have been labeled.

**Figure 4:**
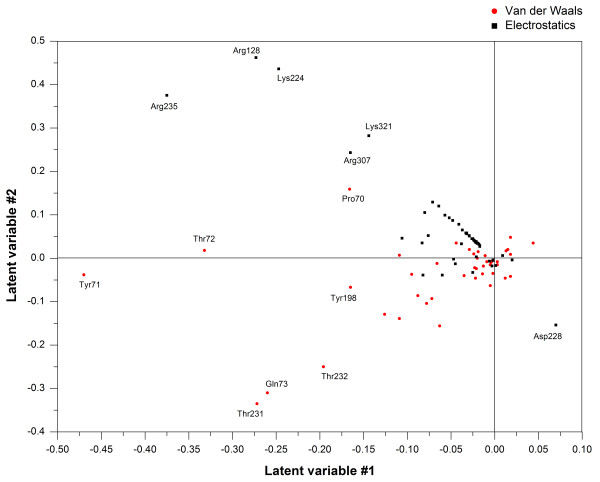
**Loading plot of the first (PC1) and the second (PC2) principal components for COMBINE.** The relevant energy descriptors have been labeled.

The essential data patterns can be easily visualized by plotting the complexes in the space defined by the first and second PCs (score plot), and the score plot of the first two principal components (PC1 and PC2) is shown in Figure 3. Alternatively, the relationship between the original variables and the new orthogonal latent variables can be revealed by plotting the contributions of the calculated energy descriptors to each of these PCs (loading plot), and the loading plots are shown in Figure 4.

As can be seen in Figures 3 and 4, the first PC extracted, which consists primarily of the van der Waals contributions involving Tyr71, Thr72, Thr231 and Gln73 and the electrostatic contributions from Arg235, Arg128 and Lys224, is sufficient to classify 38 inhibitors (training set) into two groups. One group was primarily composed of aminopyridine analogues, which are characterized by low affinity and low molecular weights, whereas the other group was mainly composed of high-molecular-weight compounds, including some peptidomimetic inhibitors. The second PC, with major contributions from the van der Waals interactions involving Thr231, Gln73 and Thr232 and the electrostatic contributions involving Arg128, Lys224, Arg235 and Lys321, is also able to distinguish the 38 inhibitors of the training set into two groups, similar to the first PC. In addition to the two groups mentioned above, there are some outliers scattered in the lower right portion of the quadrant in the score plot (Figure 4), such as the eight-residue transition-state inhibitors 45 and 46 (OM99-2[52] and OM00-3 [53]), which fill all eight binding subsites (from S4 to S4').

The most powerful variable in the first PC is the van der Waals interaction energy of Tyr71. A favorable interaction with Tyr71 is observed for inhibitors 45 and 46 (OM99-2 and OM00-3), whereas unfavorable interactions are observed with the aminopyridine analogues in the first principal component. This observation can be explained as follows: Tyr71 is a flap residue that occupies the S_1_ pocket. When analyzed by the X-ray complexes, the FLAP loop was observed to be in an open conformation when BACE-1 bound to the low molecular weight aminopyridine analogues. This conformation results in weak van der Waals interactions between the inhibitors and Tyr71. However, when bound to high-molecular-weight inhibitors such as OM99-2 and OM00-3, the FLAP loop was in a closed conformation, which indicates that strong van der Waals interactions occurred between the inhibitors and Tyr71. Note that Tyr71 is involved in a chain of hydrogen bonds with several residues in the binding pocket [46], thus fixing the flap in a closed conformation upon binding of a high-affinity inhibitor.

**Figure 5:**
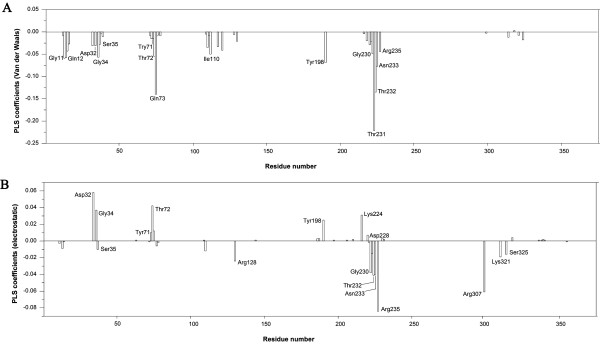
Normalized PLS coefficients for each of the (A) van der Waals and (B) electrostatic interaction energies studied.

**Figure 6:**
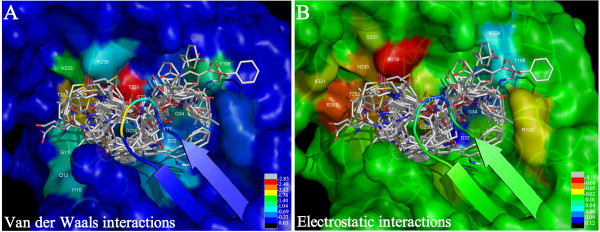
**Forty-six inhibitors were superimposed into the active site of BACE-1 (1 W51 structure).** The semitransparent surface enveloping the BACE-1 target has been spectrum-colored using the van der Waals **(A)** and electrostatic **(B)** PLS coefficients from the fourth column (B-factor) in the PDB file generated by gCOMBINE. A color scale is provided in the bottom-right corner of the both figures.

**Figure 7:**
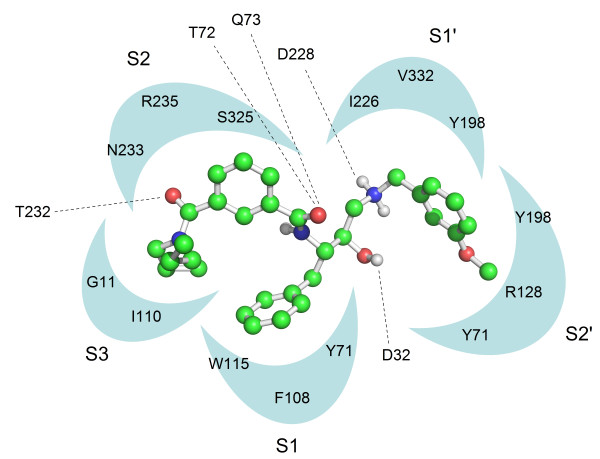
Schematic representation of the main interactions of compound 1 with the BACE-1 catalytic site.

It is apparent from the COMBINE analysis that only a limited number of interactions have a strong influence for most of the binding differences observed among the BACE-1 inhibitors. Although BACE-1 contains 375 amino acid residues, a large portion of these residues were not considered in the COMBINE analysis. The normalized PLS coefficients can quantitatively and rapidly help us to understand the different ligand-residue interactions that result in different activities (pIC_50_). According to Eq 2, the pIC_50_ values are mainly determined by the large PLS coefficients, w_i_, and the large interaction energies, u_i_. The normalized PLS regression coefficients in the first three latent variables (LV1-3) are shown in Figure 5, and the PLS regression coefficients can be color-coded and displayed on a surface representation of the protein, as shown in Figure 6. In addition, Figure 7 indicates the main interactions of compound 1 with the BACE-1 “key” amino acid residues named in this work. In order to investigate the RMSD between the ‘open’ and ‘closed’ conformations of BACE-1, based on main chain conformations, the 46 ligand-bound X-ray structures could be formed four distinct clusters. From which, 1W51, 1FKN, 2OHL, and 2OHS were chosen and superimposed together (Additional file 2: Figure S2).

We used a threshold of 0.05 on the PLS coefficients to extract important van der Waals variables with large PLS coefficients and a threshold of 0.02 on the PLS coefficients for the important electrostatic variables. With respect to the van der Waals block, it can be seen in Figure 5A that there are negative PLS coefficients for Gly11, Gln12, Gly34, Thr72, Gln73, Ile110, Tyr198, Thr231, Thr232 and Asn233, indicating that favorable van de Waals interactions with these residues are beneficial for activity. These residues form hydrophobic pockets (S_1_, S_2_, S_3_ sp, S_1’,_ S_2’_) to accommodate the substituents of the inhibitors. Of all of these ligand interactions, Thr231 appears to be the most discriminatory for activity. As mentioned above, after the X-ray complexes were superimposed, some differences emerged in the side-chain conformations of Thr231, Thr232 and Gln73, which indicates that these residues can move and rotate, with the most notable movement occurring for Gln73, to better accommodate the inhibitors. Additionally, some expected flexibility was also observed in various residues lining the binding site cleft such as Arg128, Arg307 and Arg235.

Ile110 and Gly11 are situated in the S_3_ subpocket (S_3_ sp). The S_3_ and S_1_ pockets (Tyr71, Phe108 and Trp115) are contiguous in BACE-1, and the S_3_ pocket is rather hydrophobic in nature. Many inhibitors formed favorable contacts at this site in the S_3_ pocket, and this location was considered to be a useful area to target in drug design. The extension of this pocket primarily depends on the conformation of the 10S loop. From the comparative analysis of the BACE-1 X-ray structures, it is clear that the 10S loop, a short loop located at the base of the S_3_ sp, displays two primary low-energy conformations: an open conformation (e.g., 1W51, 1TQF, 2F3E and 2B8V) and a closed conformation (e.g., 1FKN, 1M4H and 1XS7).

Tyr198 is located in the hydrophobic S_2’_ pocket (Tyr198, Tyr71 and Arg128) and the hydrophobic S_1’_ pocket (Tyr198, Ile226 and Val332). We found that the P_2’_ moiety of some inhibitors entirely fills the S_2’_ pocket with a benzyl ring. Because of its limited dimensions, the hydrophobic S_1’_ pocket only appears to tolerate an alkyl or cycloalkyl chain with a maximum of three carbon atoms. This position was employed to achieve selective BACE inhibition and should be investigated further.

It is worth noting that Tyr71 has a small negative PLS coefficient, highlighting the fact that the van der Waals interactions with this residue are slightly correlated with activity. However, in the first principal component, this residue is the most important van der Waals interaction energy variable. The PLS analysis seeks variables that can provide effective discrimination between weak and tight binders, and these variables do not need to be those with the greater absolute values, meaning that differences in the van der Waals interaction energies involving Tyr71 cannot be used in the chemometric method for the purposes of correlation with differences in inhibitory potency. Thus, this interaction, although important for binding, undoubtedly constitutes a relatively small contribution to the inhibitory activity.

In terms of the electrostatic block, it can be seen in Figure 5B that the negative PLS coefficients for Arg128, Gly230, Thr232, Asn233, Arg235, Arg307 and Ser325 co-existed with the positive PLS coefficients for Asp32, Gly34, Thr72, Tyr198 and Lys224, indicating that favorable electrostatic interactions with these residues are beneficial for activity.

As depicted in Figures 5 and 6, Asn233, Arg235 and Ser325, which are located in the S_2_ open region, can be either hydrophobic or polar. It was found that a sulfonamide or a hydrophobic phenyl ring of the inhibitors can interact with these surrounding residues, suggesting that more negative or more hydrophobic substituents are favorable in this region to improve the inhibitory activity. For the positively charged Arg128 residue, located in the S_2’_ pocket, inhibitors including one or more acidic groups on the P2’ branch are expected to favor the interaction with BACE-1. Most inhibitors donate a hydrogen bond to the backbone carbonyl of Gly230, which is located on the edge of the S_3_ pocket as shown in Figures 6 and 7. Upon comparing the binding modes of the aligned inhibitors, we noticed that hydrogen bonds with the backbones of Gly230, Thr72, or Gln73 are frequently present, and this interaction appears to be vital for high BACE-1 inhibitory activity. The aforementioned S_3_ sp, Thr232 and Arg307 are additional points of ligand attachment, which have negative PLS coefficients. Compounds presenting a polar interaction with side-chains of these residues can strengthen the inhibitor binding.

Alternatively, in the catalytic region, which was assigned by the PLS model to an electrostatic interaction, the catalytic residues (Asp32 and Asp228) are assigned positive coefficients. This result means that a positive value for the electrostatic interaction energy of the inhibitor with these two particular amino acids will favor binding, suggesting that the inhibitor with a more positive substituent in the catalytic region would increase the inhibitory potency. In the 46 X-ray protein/ligand complexes, all of the inhibitors except 32P (1TQF) formed a hydrogen bond with Asp32 or Asp228.

The abovementioned Tyr198 residue, with a hydroxyl group, is assigned a positive coefficient. Thus, the positive substituents of the inhibitors should strengthen the binding of these inhibitors. Gly34, with a backbone carbonyl, and Thr72, with a hydroxyl group, are assigned positive coefficients. The positive substituents of the inhibitors should strengthen the binding of these inhibitors. The region (Lys224) between the S1 and S1’ pockets also results in positive coefficients, suggesting that this is another area where more positively charged substituents of the inhibitors can interact.

## Conclusion

In the present study, we report an application of one of the newer 3D QSAR methods developed by A. R. Ortiz [30]. COMparative BINding Energy (COMBINE) [31-37] to a data set of 46 X-ray co-crystallized inhibitors of BACE-1. Based on the binding conformations obtained by superimposing 46 X-ray protein/ligand complexes, two predictive and robust COMBINE models were developed by correlating the pIC_50_ values with the van der Waals and electrostatic interactions that exist between the inhibitors and each protein residue. The reliability of the models was verified by the inhibitors in the testing set. The two models develop were (i) a 3-PC distance-dependent dielectric constant model (built from a single X-ray crystal structure) with a q^2^ value of 0.74 and an SDEC value of 0.52; and (ii) a 5-PC sigmoidal electrostatic model (built from the actual complexes present in the PDB) with a q^2^ value of 0.79 and an SDEC value of 0.41.

The conventional 3D-QSAR approaches, such as CoMFA and CoMSIA [41], are limited because their prediction functions rely solely on the physico-chemical parameters of substituents in a congeneric series of compounds or on molecular interaction fields calculated at discrete points in a three-dimensional (3D) lattice. Moreover, they cannot provide information concerning protein-ligand interactions. The COMBINE method used in the present study can offer detailed information describing the protein-ligand interactions and serve as a QSAR model to assess the activity of the compounds. In our 3-PC COMBINE model, the differences in the inhibitory activity of the set of inhibitors are primarily due to the van der Waals interactions with Tyr71, Gln73, Ile110, Tyr198, Thr231, Thr232 and Asn233 and the electrostatic interactions with Asp32, Gly34, Thr72, Arg128, Tyr198, Gly230, Thr232, Asn233, Arg235, Arg307 and Ser325. Thus, a total of 15 active-site residues of the receptor may be vital for ligand binding. Accordingly, strong inhibitors should have structural features that participate in favorable interactions with these protein residues. These residues are important for fine-tuning the inhibitory potency.

In our study, we did not investigate the electrostatic desolvation effects computed with a Poisson-Boltzmann model, which has been proven to yield improved COMBINE models in several previous studies [31,33,54,55]. Nevertheless, our COMBINE models provided useful insights that can be used to design novel BACE-1 inhibitors for the treatment of Alzheimer’s disease.

## Methods

### Data set

The data set used for the QSAR analysis contains 46 BACE-1 inhibitors. All of these inhibitors are ligands that were co-crystallized with the enzyme and belong to structurally different classes that were selected from the literature so as to maintain the spread of biological activity and structural diversity within and between the series. These molecules are derivatives of the following classes: eight-residue transition-state inhibitors [52,53], statine-based core structures [56], hydroxyethylamines [57-60], hydroxy ethylamines [61], hydroxyethyl secondary amine isosteres [62], isophthalamides [63,64], aminoethylene tetrahedral intermediate isosteres [65], cycloamide–urethanes [66], macrocyclics [67], macroheterocyclics [68], macrocyclic tertiary carbinamines [69], aminoheterocycles [28], piperidines [28], aliphatic hydroxyls [28], isonicotinamides [70], oxadiazoyl tertiary carbinamines [71], spiropiperidine iminohydantoins [72], piperazinones [73], imidazolidinones [73], acylguanidines [74], ψ[CH_2_NH] reduced amide isosteres [75], 1,3,5-trisubstituted aromatic [27], pyrrolidines [76] and piperidines [76]. Based on the Tanimoto coefficient using the ‘selector’ utility in SYBYL software (version 8.1) [77], these molecules were found to meet the structural diversity requirements. The 46 X-ray structures of BACE-1/inhibitor complexes used in this study are 1W51, 1TQF, 1YM2, 1YM4, 2B8V, 2F3E, 2F3F, 2IQG, 2IRZ, 2IS0, 2OAH, 2OHL, 2OHM, 2OHP, 2OHQ, I2OHR, 2OHS, 2OHT, 2OHU, 2P83, 2PH6, 2B8L, 2QZL, 2ZE1, 2QP8, 2VIE, 2VJ7, 2VJ9, 2VNM, 2VNN, 2WF0, 2WF1, 2ZDZ, 2FDP, 3CIB, 3CIC, 3CID, 3DUY, 3DV1, 2P4J, 3FKT, 2QK5, 1XS7, 1FKN and 1M4H. All of these structures were retrieved from the Brookhaven PDB [42].

The inhibitory activity data were obtained from the BindingDB database [78]. IC_50_ values are available for most inhibitors except for the complexes 1FKN, 1M4H, 1XS7 and 2FDP, for which IC_50_ values were calculated from K_i_ values using the Cheng-Prusoff equation [79,80]:

(1) $$\Delta \text{Gbind}={\text{RTlnK}}_{\text{i}}=\text{RTln}\left({\text{IC}}_{50}+0.{5\text{C}}_{\text{enzyme}}\right)\approx {\text{RTlnIC}}_{50}$$

R is the ideal gas constant, T is the temperature in K and C_enzyme_ is the concentration of the enzyme. In kinetic studies of BACE-1 and test BACE-1 inhibitory effects of some small molecules, the concentration of BACE-1 was 10 or 20 nM [81]. In practical applications, this concentration is negligible and can be omitted.

The IC_50_ values were converted to negative logarithmic values (i.e., pIC_50_), which range from 2.7 to 9.5, a range of almost seven orders of magnitude. Table 1 lists the molecules used in this study along with their experimental pIC_50_ values.

### Inhibitors alignment

Before superimposition, each of the 46 crystal structures was inspected, the best quality chain and the co-crystallized ligand were selected if the crystal structure has multiple chains, and the other chains were removed. In addition, except for the water molecules located in the active site, all other water molecules and cofactors were removed from the crystal structures. For alignment, we translocated the other 45 co-crystallized ligands into the binding pocket of 1 W51 by three protocols. **Protocol 1**. We **did not** refine the other 45 co-crystallized ligands inside their respective protein, and translocated them directly to the binding pocket of 1 W51 by a superimposition method using the C_α_ atoms (with the 1 W51 structure as the reference [60]). Using the program Accelrys DS viewer (version 1.7, Accelrys Inc.) [45], the 46 co-crystallized ligands of BACE-1 were automatically put into the binding pocket of 1 W51 (Figure 6). Subsequently, each BACE-1/inhibitor complex (each inhibitor and A chain of 1 W51) was energy minimized using the AMBER 9.0 program [82]. Following the completion of the molecular alignment processes, each inhibitor conformation in the binding pocket was individually inspected. **Protocol 2**. We **did** refine the other 45 co-crystallized ligands before translocation and then used the same method discussed in protocol 1 to align the molecules before energy minimization was performed. **Protocol 3**. We used a docking method to translocate the other 45 co-crystallized ligands to the binding pocket of 1 W51 for alignment, followed by the same energy minimization approach. Docking experiments were performed using the Surflex program [83,84] with an empirical scoring function (based on the Hammerhead docking system). The empirical scoring function has been updated and re-parameterized with additional negative training data along with a search engine that relies on a surface-based molecular similarity method. Standard parameters were used as implemented in the SYBYL software (version 8.1) [77]. The search strategy of Surflex employs an idealized ligand (called protomol), which uses various molecular fragments. Molecular fragments were tessellated in the active site and optimized based on the scoring function. The search algorithm uses the morphological similarity function, which is evaluated between the protomol and the putative ligands. For the docking algorithms, a post-dock minimization procedure was applied using the BFGS quasi-Newton method and an internal Dreiding force field. For each compound, the top 30 ranked poses were saved.

### Parameterization of complexes

The parametrization was performed using the xLEaP module of the AMBER9.0 program [82]. The all-atom AMBER 1994 force-field parameters were assigned to the protein atoms [85]. The aspartate residues located in the active site were adjusted to an ideal protonation state (Asp32 was protonated, whereas Asp228 was ionized) based on previous studies [20,43,46].

Each of the 46 BACE-1 inhibitors was assigned AM1-BCC charges and fully optimized at the AM1 level using the MOPAC 6.0 program [86]. The ligand structures were modified using the antechamber suite of the AMBER program to create input files that could be read by Leap to generate the parameter and topology files. The antechamber suite has been developed to be used with the general AMBER force field (GAFF) for small molecules [87].

### Energy minimization of complexes

For comparison purposes, we not only performed the energy minimization on the BACE-1/inhibitor complexes (each inhibitor and the A chain of 1W51) with the above-mentioned protocols, but also applied a similar energy minimization approach on the 46 complexes present in the PDB. The generalized Born (GB) continuum model for the solvation free energy is a fast and accurate alternative to an explicit solvent model for molecular simulations [88]. The GB model corresponding to igb = 5 in the AMBER 9.0 program was used. Each BACE-1/inhibitor complex was energy minimized in a sequential manner. First, the hydrogen positions were refined with 1000 steps of steepest descent energy minimization. Then, the entire system was optimized with 2000 steps of steepest descent and 3000 steps of conjugate gradient energy minimizations. The convergence criterion was that the root-mean-square value of the Cartesian elements of the energy gradient was less than 10^−2^ kcal/(mol·Å). A nonbonded cutoff of 10.0 Å and a distance-dependent dielectric constant (ε = 4r_ij_) were used. This rather conservative minimization protocol was deemed sufficient to account for the minor conformational adjustments reported in the formation of the various complexes.

### COMBINE analysis

The gCOMBINE program [38] (provided by A. Morreale) was used to decompose the interaction energy between the inhibitor and the protein in each minimized complex. That is, this program was used to calculate the Lennard-Jones and electrostatic interactions between the inhibitor and each protein residue. gCOMBINE is a GUI based on Java Swing, and the required external libraries, which are composed primarily of the command-line COMBINE program (provided by A. R. Ortiz) [30] for the algorithm of COMBINE, can be found in many articles [31-37].

In the first step of the COMBINE analysis, a set of structures of receptor-ligand complexes was prepared and the total binding energy was calculated for each of these complexes. The following step was the decomposition of the receptor-ligand interaction energy on a per residue basis for each of the complexes. An X matrix was then constructed in which the rows represent the different compounds studied and the columns contain the residue-based energy information, which is separated into two blocks (van der Waals and electrostatic), plus an additional column containing the experimental binding affinities. This X matrix was then projected onto a small number of orthogonal latent variables (PCs) using partial least-squares (PLS) analysis, and the original energy terms were given weights, w_i_, according to their importance in the model, in the form of PLS pseudocoefficients. The higher these coefficients are, the more significant they are for explaining the variance in the experimental data. Thus, in this study, the van der Waals interactions, u_i_^vdw^, and the electrostatic interactions, u_i_^ele^, between the inhibitor and each protein residue in the energy-minimized structures of the BACE-1/inhibitor complexes were selected to estimate the pIC_50_ value:

(2) $$pIC50=\sum_{i}w_{i}^{\mathrm{vdw}}u_{i}^{\mathrm{vdw}}+\sum_{i}w_{i}^{\mathrm{ele}}u_{i}^{\mathrm{ele}}+C$$

The important residues contributing to the activity should exhibit large w_i_^vdw^ and/or w_i_^ele^ values. The variables that were unimportant for activity were discarded and the remaining variables were used to build the final PLS model.

Since there are 375 amino acids in the protein and two energy contributions (van der Waals and electrostatic) are considered for each residue, 750 variables were used to characterize each complex. These energy descriptors comprised the matrix for the gCOMBINE program. No scaling or variable selection was performed except for a mild pretreatment that consisted of zeroing all the variables with absolute values lower than 0.01 kcal/mol and removing those variables with a standard deviation below 0.01 kcal/mol. This procedure reduced the number of energy descriptors that entered the PLS analysis. The optimal dimensionality of the PLS models was determined by monitoring the cross-validation indexes as a function of the number of principal components (PCs) extracted. The cross-validation procedure employed the leave-one-out method. The predictive ability of the resulting models was reported by both the cross-validated correlation coefficient (q^2^) and the standard deviation of error in predictions (SDEP).

Followed by energy minimization, for comparison purposes, we not only performed COMBINE analysis on the BACE-1/inhibitor complexes (each inhibitor and the A chain of 1W51), but also performed COMBINE analysis on the actual complexes present in the PDB after a similar energy refinement. To guarantee the COMBINE analysis is successful, it is important to ensure that the protein is exactly the same for all complexes and that all the residues are exactly the same. In addition, the same active site accommodating the various ligands must be identical for each complex. In the present study, we found that the number of amino acids differed in some complexes; therefore we applied a common number of residues for all the complexes ("normalization") to remedy this problem.

To study the robustness of the above procedure, the complexes in the prediction sets were determined as follows. In the complete data set, the pIC_50_ values varied from 2.7 to 9.5, and therefore, the complexes were classified into seven activity ranges from 2.5 to 9.5 using increments of 1.0. One randomly chosen complex per range was assigned to the prediction set, but two complexes were chosen from the ranges [3.0−4.0] and [4.0−5.0] because these two ranges contained the majority of the complexes. As a result, a total of eight randomly chosen complexes (inhibitors **2, 13,15, 21,36, 40,42** and **44**) were included in the prediction set.

## Competing interests

The authors declare that they have no competing interests.

## Authors' contributions

Shu Liu and Li-Hua Zhou conceived of the study; Shu Liu designed the experiment; Rao Fu and Xiao Cheng performed the calculation; Sheng-Ping Chen contributed data analysis; Shu Liu and Rao Fu Wrote the paper. All authors read and approved the final manuscript.

## Supplementary Material

Additional file 1 **Figure S1.** Data set of the 46 co-crystallized ligands of BACE-1 (Elarged pictures) Data set of the 46 co-crystallized ligands of BACE-1. Click here for file

Additional file 2 **Figure S2.** Cartoon representation of the active site of the four BACE-1 X-ray structures used for the superposition study (1W51, 1FKN, 2OHL, and 2OHS are in green, cyan, magenta and yellow, respectively) with compound 1 shown as ball and sticks; hydrogen atoms are omitted for reasons of clarity. Click here for file
