# Supplementary material for: Exploring the binding of BACE-1 inhibitors using comparative binding energy analysis (COMBINE)
Source: BMC Struct Biol. 2012 Aug 27;12:21. doi: 10.1186/1472-6807-12-21 (PMC3533579; doi:10.1186/1472-6807-12-21)
Supplement: Additional file 1 — Figure S1. Data set of the 46 co-crystallized ligands of BACE-1 (Elarged pictures) Data set of the 46 co-crystallized ligands of BACE-1. [file 1472-6807-12-21-S1.docx]

**Figure S1 Data set of the 46 co-crystallized ligands of BACE-1 (Elarged pictures)**

|  |
| --- |
| ^a^1. 1W51, L01 |
|  |
| 2. 1TQF, 32P |
|  |
| 3. 1YM2, AUA |
|  |
| 4. 1YM4, AMK |
|  |
| 5. 2B8V, 3BN |
|  |
| 6. 2F3E, AXQ |
|  |
| 7. 2F3F, AXF |
| **** |
| 8. 2IQG, F2I |
|  |
| 9. 2IRZ, I02 |
|  |
| 10. 2IS0, I03 |
|  |
| 11. 2OAH, QIN |
|  |
| 12. 2OHL, 2AQ |
|  |
| 13. 2OHM, 8AP |
|  |
| 14. 2OHP, 6IP |
|  |
| 15. 2OHQ, 7IP |
|  |
| 16. 2OHR, 8IP |
|  |
| 17. 2OHS, 9IP |
|  |
| 18. 2OHT, IP6 |
|  |
| 19. 2OHU, IP7 |
|  |
| 20. 2P83,MR0 |
|  |
| 21. 2PH6, 712 |
|  |
| 22. 2B8L, 5HA |
|  |
| 23. 2QZL, IXS |
|  |
| 24. 2ZE1, 411 |
|  |
| 25. 2QP8, SC7 |
|  |
| 26. 2VIE, VG0 |
|  |
| 27. 2VJ7, VG6 |
|  |
| 28. 2VJ9, VG7 |
|  |
| 29. 2VNM, CM8 |
|  |
| 30. 2VNN, CM7 |
|  |
| 31. 2WF0, ZY0 |
|  |
| 32. 2WF1, ZY1 |
|  |
| 33. 2ZDZ, 310 |
|  |
| 34. 2FDP, FRP |
|  |
| 35. 3CIB, 314 |
|  |
| 36. 3CIC, 316 |
|  |
| 37. 3CID, 318 |
|  |
| 38. 3DM6, 757 |
|  |
| 39. 3DUY, AFJ |
|  |
| 40. 3DV1, AR9 |
|  |
| 41. 2P4J, 23I |
|  |
| 42. 3FKT, SII |
|  |
| 43. 2QK5, CS5 |
|  |
| 44. 1XS7, MMI |
|  |
| 45. 1FKN, OM99-2 |
|  |
| 46. 1M4H, OM00-3 |
