# Supplementary material for: Exploring the binding of BACE-1 inhibitors using comparative binding energy analysis (COMBINE)
Source: BMC Struct Biol. 2012 Aug 27;12:21. doi: 10.1186/1472-6807-12-21 (PMC3533579; doi:10.1186/1472-6807-12-21)
Supplement: Additional file 2 — Figure S2. Cartoon representation of the active site of the four BACE-1 X-ray structures used for the superposition study (1W51, 1FKN, 2OHL, and 2OHS are in green, cyan, magenta and yellow, respectively) with compound 1 shown as ball and sticks; hydrogen atoms are omitted for reasons of clarity. [file 1472-6807-12-21-S2.docx]

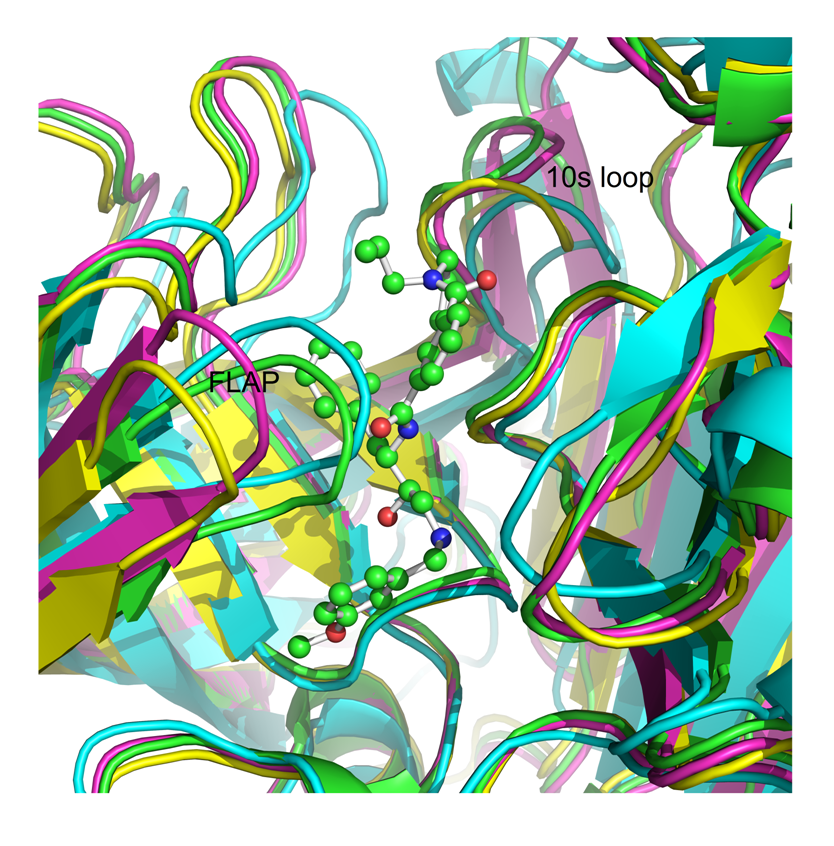


**Figure** S2. Cartoon representation of the active site of the four BACE-1 X-ray structures used for the superposition study (1W51, 1FKN, 2OHL, and 2OHS are in green, cyan, magenta and yellow, respectively) with compound 1 shown as ball and sticks; hydrogen atoms are omitted for reasons of clarity.
